# Consequences of Arsenic Contamination on Plants and Mycoremediation-Mediated Arsenic Stress Tolerance for Sustainable Agriculture

**DOI:** 10.3390/plants11233220

**Published:** 2022-11-24

**Authors:** Anmol Gupta, Priya Dubey, Manoj Kumar, Aditi Roy, Deeksha Sharma, Mohammad Mustufa Khan, Atal Bihari Bajpai, Ravi Prakash Shukla, Neelam Pathak, Mirza Hasanuzzaman

**Affiliations:** 1 IIRC-3, Plant-Microbe Interaction and Molecular Immunology Laboratory, Department of Biosciences, Faculty of Science, Integral University, Lucknow 226026, Uttar Pradesh, India; 2 CSIR-National Botanical Research Institute, Rana Pratap Marg, Lucknow 226001, Uttar Pradesh, India; 3 Institute of Plant Sciences, Agricultural Research Organization, Volcani Center, Rishon LeZion 7505101, Israel; 4 Plant Molecular Biology Laboratory, CSIR National Botanical Research Institute, Lucknow 226001, Uttar Pradesh, India; 5 Department of Basic Medical Sciences, Integral Institute of Allied Health Sciences & Research (IIAHS&R), Integral University, Lucknow 226026, Uttar Pradesh, India; 6 Department of Botany, D.B.S. (PG) College, Dehradun 248001, Uttarakhand, India; 7 Village and Post-Barahuwan, Basti 272123, Uttar Pradesh, India; 8 Department of Biochemistry, Dr. Rammanohar Lohia Avadh University, Ayodhya 224001, Uttar Pradesh, India; 9 Department of Agronomy, Faculty of Agriculture, Sher-e-Bangla Agricultural University, Dhaka 1207, Bangladesh

**Keywords:** arsenic, metalloid toxicity, oxidative stress, signal transduction, mycoremediation, stress tolerance

## Abstract

Arsenic contamination in water and soil is becoming a severe problem. It is toxic to the environment and human health. It is usually found in small quantities in rock, soil, air, and water which increase due to natural and anthropogenic activities. Arsenic exposure leads to several diseases such as vascular disease, including stroke, ischemic heart disease, and peripheral vascular disease, and also increases the risk of liver, lungs, kidneys, and bladder tumors. Arsenic leads to oxidative stress that causes an imbalance in the redox system. Mycoremediation approaches can potentially reduce the As level near the contaminated sites and are procuring popularity as being eco-friendly and cost-effective. Many fungi have specific metal-binding metallothionein proteins, which are used for immobilizing the As concentration from the soil, thereby removing the accumulated As in crops. Some fungi also have other mechanisms to reduce the As contamination, such as biosynthesis of glutathione, cell surface precipitation, bioaugmentation, biostimulation, biosorption, bioaccumulation, biovolatilization, methylation, and chelation of As. Arsenic-resistant fungi and recombinant yeast have a significant potential for better elimination of As from contaminated areas. This review discusses the relationship between As exposure, oxidative stress, and signaling pathways. We also explain how to overcome the detrimental effects of As contamination through mycoremediation, unraveling the mechanism of As-induced toxicity.

## 1. Introduction

Arsenic (As) is extensively recognized as a human carcinogen worldwide and declared as a non-threshold toxic pollutant that is a toxic metalloid [1]. It occurs in two forms: inorganic-like arsenate (AsV), arsenite (AsIII), and organic forms, for instance, dimethyl arsenic acid (DMA), monomethylarsonic acid (MMA), trimethylarsine oxide, and trimethyl arsine (TMA). The inorganic form of arsenic has much more toxicity than the organic form. The natural sources of As pollution in water and soils include weathering of rocks, volcanic activities, and major anthropogenic activities, including applying pesticides, mining, and paints, among others [2]. High As concentrations are predominantly measured in drinking water found in vast areas of West Bengal (India) and Bangladesh, and smaller areas of the USA, Argentina, Mexico, Chile, Taiwan, Australia, and Vietnam (IARC, 2012). In some regions of Mexico, USA, Brazil, Japan, Thailand, and Australia, mining, smelting, and other industrial activities have contributed to the upliftment of As in nearby water bodies, thereby affecting the local areas (IARC, 2004). Various anthropogenic sources (such as cropland treated with As pesticides and mine waste) can have high As concentrations in soil ranging from 5 mg kg^−1^ to 3000 mg kg^−1^ (WHO, 2001). However, due to its high transfer capacity, As is easily spread from water to soil, soil to water, and then to various crops [3,4]. The contamination of As in water and soil is becoming an irreversible problem and causing a serious hazard to human health, causing several diseases such as cardiovascular, skin cancer, and neurological disorders [5,6]. Arsenic not only restricts plant development and soil fertility but also disrupts the food chain and food web [2]. Since As persist in nature for a long time, it is necessary to understand its toxic effect on health [3]. In the opinion of the Food and Agriculture Organization and the World Health Organization (FAO and WHO), the concentration of As in soil should be below 20 mg kg^−1^, while for paddy grains it should be less than 1 mg kg^−1^ dry weight.

Many living organisms are present near the healthy soil, forming a large creature of fungi, bacteria, earthworms, algae, protozoa, nematodes, among others. These beneficial microbes are very useful for the bioremediation of toxic metals. Metal-tolerant microorganisms can persist in polluted areas and be utilized for bioremediation purposes [7,8]. Microorganisms have evolved various biochemical mechanisms to feed the As oxyanions, either as an electron donor or an electron acceptor (AsV) for anaerobic respiration and to support the chemo-autotrophic fixation of carbon dioxide (CO_2_) into the cell carbon [9]. Among them, fungi have a great capacity for As detoxification because of their prevalence of large biomass in the soil and longer life cycle [10]. The toxic metals can also be removed or fixed via the mycoremediation process. In the mycoremediation process, As-resistant fungi are used to degrade or sequester As in the contaminated area by using its enzymatic activities [11,12]. The fungal cell wall has specific metal-binding peptides, proteins, and polysaccharides that contain hydroxyl (HO^−^), carboxyl (R-COOH), phosphate (PO_3^−^_), sulfate (SO_2^4^−^^_), and amino (NH_2_) groups that bind metalloid ions which are used for mycoremediation purposes [7,13,14,15,16]. Bioaccumulation and biomethylation have been recommended as detoxification mechanisms for microorganisms that occur in As-contaminated matrices [17]. Fungi have been identified as promising cost-effective adsorbents for As removal from the polluted area. Most of the fungal strains, such as *Saccharomyces cerevisiae* [18], *Trichoderma* sp. [19], *Penicillium* sp., [20] *P. verrucosum* [21], *Ascomycota*, and *Basidiomycota* [11], *Aspergillus flavus (FS4)* and *A. fumigatus* [22], *Aspergillus* sp. [23,24], *A. versicolor* [25,26], *Rhizopus* sp. [27], and *Metarrhizium anisoplia* [28,29] have been broadly studied as potential microbial agents for the elimination of toxic metals from the polluted area. Therefore, bioaccumulation and metal chelation by fungus can be used to treat metal-containing wastewater at low cost.

## 2. Effects of Arsenic Contamination on Microbial Dynamics and Crops

In soil, As is present in numerous forms owing to its interactions with various components available in soil. Hence, the total As concentrations in soil cannot provide a defined index for evaluating the soil microflora and their enzymatic activities [30]. The functional groups of microbes bind with As, which is present on the cell wall and cell membrane, thereby binding with proteins, PO_3^−^_, and HO^−^ groups of nucleic acids such as DNA/RNA [12]. This leads to impairing of functions and causes the protein to denature, thereby inhibiting the cell division which is the most substantial part of microbial growth.

**Figure 1:**
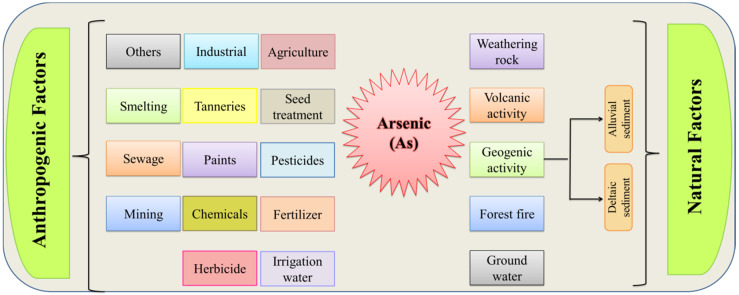
Sources of arsenic contamination in soil.

Arsenic is harmful to various crops; even its minimum quantity causes a diversity of toxic effects on plants. Plants can also be affected by As through stunted roots, withered leaves, reductions in photosynthetic pigment, yellowing of leaves, and reduced chlorophyll (Chl), thus affecting plant metabolism [31,32]. Arsenic levels are typically modest in plants growing in natural soil (3.6 mg kg^−1^) [33]. Most plants are harmed by As at greater concentrations. Arsenic-induced phytotoxicity is led to interfering with many metabolic processes, thereby inhibiting the plant growth and development of particular crops. However, there are certain circumstances where the terrestrial vegetation may accumulate the As uptake by roots from soil or through airborne absorption. There are various natural and anthropogenic factors that cause As contamination in soil (Figure 1). Arsenate is a more dominant species in the soil, and on account of its similarity with PO_3^−^_, it is further competing for the carriers’ uptake in the root plasma-lemma. Among the toxic symptoms studied in this study, the inhibition of seed germination was the most notable [34]. In their study, Khanna et al. [35] suggested that it is crucial to quantify photosynthetic pigments such as chl a and chl b contents in rice leaves to show their correlation with rice yield. Paddy rice is more susceptible to As deposition than any other crop due to its great mobility under flooded conditions. It is well reported in a previous study that arsenic toxicity causes a reduction in wheat crops, because of a reduction in the amylolytic activity [36]. The first species recognized as an As hyperaccumulator is *Pteris vittata*, sometimes referred to as brake fern. While the bioaccumulation of As in aquatic species mostly affects algae and lower invertebrates, brake fern may also hyperaccumulate As, producing from insoluble forms up to three to six times more than the As concentration in soil [37,38].

Duxbury and Panaullah [39] analyze that As in rice grain and soil were shown to be negatively correlated; As 0.54 mg kg^−1^ in rice at soil As 11.6 mg kg^−1^ versus 0.34 mg kg^−1^ (rice) at soil As 57.5 mg kg^−1^, while As in rice straw correlated positively with soil As. According to them, As toxicity interferes with the translocation of As from vegetative tissues to grain. In this trial, relatively high grain As contents occurred despite relatively low soil As levels, suggesting that rice can accumulate significant amounts of As at levels well below what is considered potentially toxic.

### Mechanism of Arsenic Metabolism, Transport, and Detoxification in Food Crops

Higher concentrations of As in crop soil and groundwater may result in increased crop loss and catastrophic health effects in humans. Rice grains have been shown to accumulate 2.24 mg kg^−1^ As compared to other main food crops [40]. Having molecular similarity to silicon (Si) and phosphorus (Pi), rice plants can accumulate AsIII and AsV through Si and Pi transporters. This renders a higher risk of As accumulation in rice, thus exposing humans to As toxicity. The decline in As sequestration, uptake, and transport into the vacuoles restricts the availability of As for root-to-shoot translocation, which can further lead to a decrease in its accumulation in rice (*Oryza sativa*) grains and other food crops. Therefore, a superior understanding of As metabolism, its transportation, and its detoxification can additionally assist to identify the new genetic methods in improving and developing the low grain arsenic rice. Plant roots can uptake the As from the soil and translocate it to various parts of the plants through active/passive mode. Arsenate (AsV) (a phosphate analog), is the primary As species in aerobic soil, which is carried (through Pi transporters) from soil to above-ground portions of the plants [41]. Along with transporters, various proteins, including P_i_ transporter, phosphate transporter traffic facilitator-1 (*OsPHF1*), have additionally been recommended to regulate AsV uptake. The plasma membrane intrinsic proteins (PIP) including *OsPIP2* [42,43,44] also have their function in AsIII transport [45]. In *Arabidopsis thaliana*, it was revealed that a sugar alcohol transporter in *AtINT4* or *AtINT2* (inositol transporter) is the possible target to prevent the As loading into the phloem. However, in *O. sativa*, such transporters still need to be discovered. The detoxification procedure includes the introduction of AsV into the cell, which is subsequently reduced to AsIII using glutathione as the reductant by the arsenate reductase enzyme [46]. After reduction, further detoxification of AsIII happens in the vacuole by vacuolar sequestration. Arsenite (AsIII) chelates with some sulfhydryl rich proteins such as phytochelatins (PCs) and glutathione (GSH), and metallothioneins (MTs) and forms a complex with them that gets sequestered by vacuolar transporters. Rice ABC transporter (*OsABCC1*), a vacuolar transporter, has been shown to play a role in As detoxification by sequestering AsIII to the vacuole, hence lowering AsIII accumulation in rice grains [47]. The chloroquine-resistance transporter-like transporter (*OsCLT1*), a homolog of *AtCLT1* GSH transporters (shares 63% amino acid sequence identity), is displayed to be important for reducing As accumulation in rice. *OsCLT1* is a plastid-localized transporter that is responsible for exporting the GSH and g-glutamyl cysteine into the cytosol for maintaining the GSH homeostasis and biosynthesis. Over-expression of the glutaredoxin gene maintains the GSH pool and increases As accumulation in roots, decreasing As transfer to aerial portions of the plant, primarily the grain [48]. To protect cells from As toxicity, genetic engineering techniques using glutaredoxins are also necessary [49].

Wheat is the second most important staple food grown with an annual production of over 600 million tons [50]. However, wheat has been shown to have comparatively low As concentrations due to its growth in aerobic environments and lower affinity for silica accumulation [51]. The link between As transit and accumulation in wheat crops, particularly in diverse wheat cultivars, is poorly understood. Arsenate tolerance of 57 wheat cultivars was recently shown to vary substantially, with the tolerance being mostly attributed to As retention in roots [51]. With a mean level of 0.100 mg kg^−1^, the total As content in wheat varies from 0.010 mg kg^−1^ to 0.500 mg kg^−1^ [50,52,53].

One of the most widely grown cereals worldwide is maize (*Zea mays*), usually referred to as corn. According to Marwa et al. [54], As levels in maize in Tanzania ranged from 0.01 to 0.17 mg kg^−1^. Pulses (legume seeds) are a significant dietary source of protein for Southeast Asian communities, particularly those who rely on a vegetarian diet. A study by Williams et al. [55] revealed that five different types of pulses contain inorganic (iAs) forms of arsenic. Due to their relatively low As content and low global consumption of pulses, pulses make up a small portion of the diet As intake [55]. The estimated daily dietary intake of iAs in Brazil is 0.255 g kg^−1^ body weight or roughly 9% of the BMDL0.5 of 3 g kg^−1^ body weight.

Vegetables are a significant category of the human diet, hence many publications evaluated the levels of As in various vegetables and mushrooms. After maize, rice, and wheat, the potato (*Solanum tuberosum*) is one of the world’s major crops [56]. Williams et al. [55] identified the arsenic speciation in samples of potato tubers. The samples came from Bangladesh, and the study found no identifiable organic (oAs) forms of arsenic in the species; only iAs were present. Based on samples collected from a village in West Bengal, Signes-Pastor et al. [57] reported the presence of MMA in potato tubers. According to data on As in vegetables, some vegetables can contain concentrations of As equivalent to rice, based on dry weight [50]. A study by Bhattacharya et al. [58] showed that potatoes accumulated the highest amounts of As, even higher than that of rice. The results of an analysis by Rahman et al. [59] found that total As concentrations in food crops in Malda, West Bengal ranged from 0.000 to 1.464 mg kg^−1^ dw, with potato having the highest concentration (0.456 mg kg^−1^), followed by rice grain (0.429 mg kg^−1^). The range of the total As content was 0.032 to 0.411 mg kg^−1^ dw in vegetables, 0.031 to 0.175 mg kg^−1^ dw in spices, and 0.021 to 0.145 mg kg^−1^ in fruits. In Bangladesh, As levels have been reported to differ by areas such as Jamalpur and Chandpur districts (0.070–3.990 mg kg^−1^) and Comilla, Rajshahi, and Sathkhira districts (<0.040–1.930 mg kg^−1^) [55]. Vegetables from the Domkal and Jalangi blocks in West Bengal, India, had mean As levels of 0.0212 mg kg^−1^ (0.00004–0.212 mg kg^−1^) and 0.0209 mg kg^−1^ (0.00004–0.138 mg kg^−1^), respectively [60]. Vegetables with leaves have been shown to contain greater amounts of As (0.041–0.464 mg kg^−1^) than vegetables without leaves (0.011–0.145 mg kg^−1^) [55].

Mushrooms are a significant food item due to their nutritional benefits, and their consumption has significantly expanded globally in recent years [61,62]. Wild mushrooms have the capacity to accumulate certain elements in significant amounts in their fruit bodies [63]. Seyfferth et al. [62] examined 40 samples of 12 different varieties of mushrooms from two important As, Pb, and Cd-producing areas in the US. There were variations in both As localization (fruiting body in cremini vs. hymenophore in shiitake) and overall As concentrations (cremini > shiitake). All mushroom samples, however, had an As content of less than 1 mg kg^−1^ dw. A recent investigation of As speciation in *Elaphomyces* sp. *asperulus* found that the total As content varied from 12 to 42 mg kg^−1^ dw, while in *E. muricatus* and *E. granulatus*, it varied from 120 to 660 mg kg^−1^ dw. The dominant species of As were oAs namely, the most notable MMAV (around 30% of extractable As), while the remainder is trimethylarsine oxide (TMAO, which accounts 0.3 to 28% of extractable As) and MMAIII (which accounts 0.08–0.73% of extractable As) [64]. Due to a more effective enzymatic conversion of As from iAs to oAs species, it has been discovered that mushrooms have a proportionately higher oAs content than iAs [64]. 

## 3. Arsenic Detoxification Mechanism Using as Tolerant Fungi and Its Mitigation via Glutathione Biosynthesis

The majority of species such as *Mucor hiemalis* [65], *Rhizopus micosporus* [66], *Trichoderma brevicompactum* QYCD-6 [67], *Fomitopsis meliae* [66], *Rhizophagus irregularis* [68,69], *Funneliformis mosseae* [70], *Diversispora spurcum* [70], *Rhizophagus intraradices* [71], and *Funneliformis mosseae* [71] are harmful to toxic metals. The ability of the fungus to tolerate metals enables them to thrive in surroundings that are contaminated with hazardous metals [72,73]. Mycoremediation (remediation by fungus) is a “green-clean” environmentally friendly method that has a lot of potential for use in the removal of toxic metals and organic contaminants. It has drawn a lot of interest because it offers an alternative to conventional chemical and physical methods of removing toxic metalloids and metals. Enzymatic accumulation, detoxification inside the cell via passive (diffusion) and/or active (transport systems) uptake mechanisms, exclusion by permeability barrier, adsorption on extracellular structures (cell wall, slime, capsule), efflux pumps, intra and extra-cellular precipitation, the adjustment in the cellular targets, volatilization, methylation, and chelation of metal/loids are some of the mechanisms used by fungi to tolerate and detoxify the toxic metals [74,75].

The concentration, toxicity, and bioavailability of toxic metals as well as the features of the fungus determine the reaction of the fungi respond to metal and their level of resistance [74,76]. Physico-chemical, biological, and geological variables can all have an impact on chemistry and biogeochemistry since they are complicated. Although arsine (AsH_3_) is a very hazardous inorganic arsenic species, other species usually play a bigger role in the environment because of their sensitivity to oxygen [77]. In addition to the two most prevalent inorganic forms (AsV and AsIII), some biological systems may produce the methylated arsenic compounds MMA, DMA, and TMAO from AsIII and AsV [78].

The multifunctional bio-thiol tripeptide glutathione (L-gamma-glutamyl-L-cysteinyl-glycine) is produced by two enzymes, glutathione synthase (GS) and g-glutamylcysteine synthetase (g-GCS), in two ATP-dependent processes. Following the addition of L-glycine to the C-terminal of g-GC by GS, which produces GSH, g-GCS catalyzes the binding of L-cysteine and L-glutamate to produce g-glutamylcysteine (g-GC) [79,80]. The ectomycorrhizal (ECM) fungi are protected against As toxicity by the GSH production that is activated by the intracellular aggregation of As.

**Figure 2:**
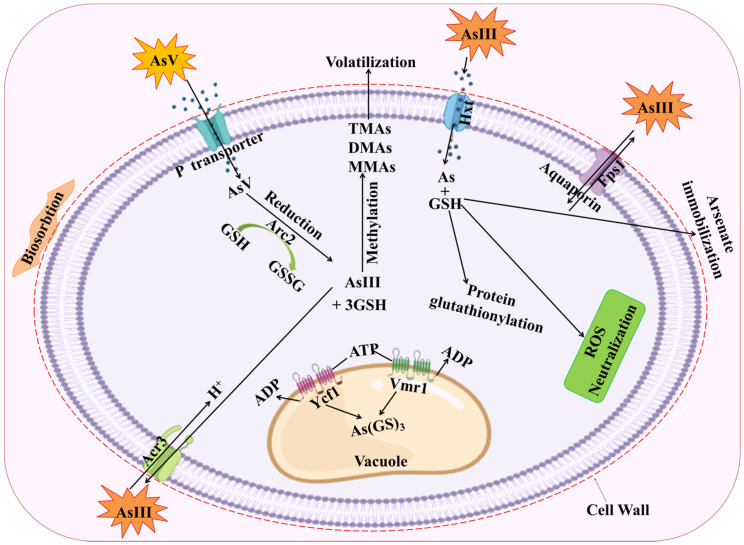
Detoxification mechanism of As toxicity via glutathione. AsV entry restricted through binding with carboxyl, amine, or other functional groups in fungal cell walls. AsV undergoes a quick enzymatic reduction to a trivalent state after entering the cytoplasm, making it a substrate for a variety of modifications and detoxification processes. Intracellular AsV is converted into AsIII, which is then transported or pumped outside of fungus cells. Arsenic is compartmentalized in the vacuole by the ABC transporters Ycf1 and Vmr1, generating As(GS)_3_. Methylation results in the production of less hazardous chemical compounds such MMAs, DMAs, and TMAs. Two genes, *ACR2* that encodes an AsV reductase and *ACR3* that encodes an AsIII exporter, may be expressed, which might trigger the tolerance to As exposure. AsIII methyltransferases are responsible for catalyzing the methylation process (*ArsMs*). The aquaglyceroporin *Fps1* primarily facilitates AsIII uptake. Aquaglyceroporin *Fps1* is primarily responsible for facilitating AsIII uptake, however AsIII can also enter cells through hexose permeases Hxt (*Hxt1-Hxt17, Gal2*) in the absence of glucose. MMAs(III)—monomethylarsonic acid; DMAs—dimethylarsonic acid; TMAs—trimethylarsonic acid; GSH—glutathione; As(GS)_3_—arsenic triglutathione.

Two high-affinity phosphate transporters, *PHO84* and *PHO89*, as well as three low-affinity phosphate transporters, *PHO87, PHO90,* and *PHO91*, have been found in some metal-tolerant yeast, and mycorrhizal fungi (*Penicillin janthinellum* SM-12F4, *Fusarium oxysporum* CZ-8F1, and *Trichoderma asperellum* SM-12F1) played a significant role in As detoxification [81,82]. Strongly enhanced AsV tolerance was seen after deletion of the *PHO84* and *PHO87* genes, indicating that the phosphate transport system in toxic metals-tolerant fungus mediates AsV absorption (Figure 2) [83,84]. Interestingly, protein alignment utilizing the sequences of yeast *PHO84* and *PHO89* indicated the presence of two different groups: H^+^:Pi transporters grouping with *PHO84* and Pi:Na^+^ transporters clustering with *PHO89* [85]. Additionally, cells inferring the phosphate transporter-associated proteins *GTR1* and *PHO88*, which control the positive transport function of *PHO84*, and the membrane protein *PHO86* is necessary for directing *PHO84* to the plasma membrane, which also shows higher resistance to AsV [75,83,84,85].

Furthermore, it has been demonstrated that yeast and metal-tolerant fungi export and accumulate tripeptide glutathione outside of cells following prolonged exposure to AsIII [86]. Fungi and yeast cells with higher extracellular GSH levels accumulate less As and show better growth when exposed to AsIII [87]. On the other hand, AsIII is sensitive to cells with defects in GSH export and extracellular accumulation. As a result, GSH is exported in this novel detoxifying pathway to protect yeast and fungal cells from AsIII toxicity [88]. Due to its function in cellular redox management, GSH not only serves as a metal chelator but also shields cells against oxidative damage brought on by metals.

The primary processes of As absorption and resistance in metal-tolerant fungus are depicted in Figure 2. Many toxic metals-tolerant fungi, including *Aspergillus* species, and yeast, *S. cerevisiae*, have an arsenate reductase called *Acr2p* that may convert AsV to AsIII, which the cell will then export outside [89]. GSH and glutaredoxin serve as an electron donor in this reaction [90]. Cells only become AsV-sensitive after *ACR2* gene deletion [90]. On certain fungi and yeast, the function of GSH in As tolerance has been shown [91,92,93]. GSH was crucial during As stress, according to comparative proteomics of As-induced differentially expressed proteins in rice plants [94]. Therefore, *Hebeloma cylindrosporum*, an ECM fungus, was used to test how GSH responded to various As concentrations. In *H. cylindrosporum*, higher As accumulation was seen to be accompanied by higher GSH concentrations. As a result of external As stress, the GSH concentration rose. This shows that when exposed to external As stress, the GSH defense mechanism is quickly triggered in ECM fungus. Numerous studies on yeast (*Candida tropicalis*, *S. cerevisiae*), fungus (*Laccaria bicolor*, *Aspergillus niger*), and other organisms have validated this conclusion [95,96,97,98,99,100]. GSH offers a two-fold defense when in contact with intracellular As. It functions as a metal scavenger as well as an antioxidant. As an antioxidant, glutathione lowers the production of free radicals brought on by arsenic stress, neutralizing their negative effects, and oxidizing itself to GSSG [101]. Pentavalent As is also converted by glutathione into trivalent As [102,103]. Further binding of glutathione results in the formation of the As-(GSH)_3_ complex, which is actively transported to vacuoles by the ABC transporters [104]. When exposed to toxic metals, different animals, plants, and fungi have been shown to produce the As-GSH conjugate [96,105]. It is important to remember, nevertheless, that the ABC proteins only function as AsIII transporters when As is complexed with the thiol group [106]. As a result, when exposed to As, the cell promptly uses the active GSH already available, inducing the GSH production process [101]. The research on ECM systems is still in its infancy, though.

Finally, it is important to emphasize that reduced GSH plays a crucial role in As tolerance and oxidative stress in yeasts and many other toxic metals tolerant fungi. Metalloids and metals in mycorrhizal fungi and yeast may bind to it in response. The proteins that regulate vacuolar sequestration use the resulting complex as a substrate. Second, a significant antioxidant is also required to counteract the ROS produced by As exposure. Finally, the process of protein glutathionylation involves the attachment of reactive sulfhydryl groups on proteins by GSH, which prevents metal binding and protein oxidation [88].

The mechanism of metal biosorption is difficult and not absolutely understood. Since recent years, several researchers are specialized in numerous aspects of biosorption mechanisms [107,108,109]. Plants, on the other hand, have a variety of As detoxification strategies, including the reduction of AsV to AsIII, which eventually form complexes with PCs, GSH, and γ-glutamylcysteine and sequestered in vacuoles [46]. The glutaredoxin, which regulates AsIII outflow, is also important for As detoxification and tolerance [93,110]. Despite the fact that AsIII is more dangerous than AsV, it will methylate to generate TMA, and the final consequence of methylation is volatile organic arsenicals, which are innocuous at low quantities [111]. Thus, As volatilization and biomethylation are considered As detoxification mechanisms in most mammals and other lower organisms [110,112]. Henceforth, biomethylation could be considered the assistance of bioremediation using microbes. Currently, bioremediation of As by various microbes is extensively used due to their possible advantages in enabling cost-effective and eco-friendly technologies. Furthermore, bioaugmentation practice could be further used to treat As-contaminated water and soil by adding genetically modified microorganisms to gather the high amount of As [113]. Moreover, biostimulation might also be used in which the organic substrates and nutrients are added to the contaminated site to augment the growth of endemic microbes which may improve the rate of As bioremediation [114,115]. Many mechanisms have been documented based on their fungal activity to immobilize As through various mechanisms such as biosorption, bioaccumulation, and biovolatilization, methylation from contaminated areas (Table 1). Removal of As pollutant by fungi (mycoremediation) is evolving as a prominent and cost-effective tool to ameliorate the As contaminants.

Mycorrhization can also increase AsV uptake by host plants since AsV is analogous to inorganic phosphate (P_i_) and uses similar phosphorus (P) transporters for entry into the plant cell [119,120]. Furthermore, ECM fungi accumulate more arsenic and prevent the transfer of As from the soil to the plant [121]. However, it is still not clear how As is detoxified and transferred to plants [96,122]. Ectomycorrhizal fungi have evolved a variety of strategies for coping with toxic metals [121,123]. In particular, these mechanisms include cellular efflux, intracellular conjugation with thiols (–SH) as MTs, and cell wall binding as well as vacuolar compartmentalization and GSH [79,91]. It has been also documented that ECM fungi respond differently to various forms of toxic metals stress [79]. According to Mukherjee et al. [98], plants have developed a reliable method for detoxifying As by conjugating it with glutathione (GSH). Arsenic is chelated by GSH by thiolate bonds and forms As(GSH)_3_ complex, which is additionally compartmentalized into vacuoles by the ABC transporters [124]. Although the significance of arbuscular mycorrhizal fungi in modulating –SH under As stress is well recognized, no further studies have been conducted, to our knowledge, to understand the role of –SH in ECM systems [92,125]. It is rare to find reports on yeast or plants which provide an analysis of GSH genes in response to As-stress and their response to it in ECM fungi [126]. Since the ECM fungus is eco-friendly and cost-efficient, understanding its mechanisms for mitigating arsenic toxicity becomes increasingly important. This review describes that amongst metal detoxification mechanisms of ecto-mycorrhizae fungi, and GSH biosynthesis which is the crucial mechanism that gets persuaded under As-stress and thus protects the plants from As toxicity. Research has been conducted in genomics, metabolomics, and proteomics to determine mechanisms that scavenge As toxicity in a variety of vegetation, including rice [94,127,128,129,130].

## 4. Mycorrhizae-Based Mitigation of Arsenic

**Figure 3:**
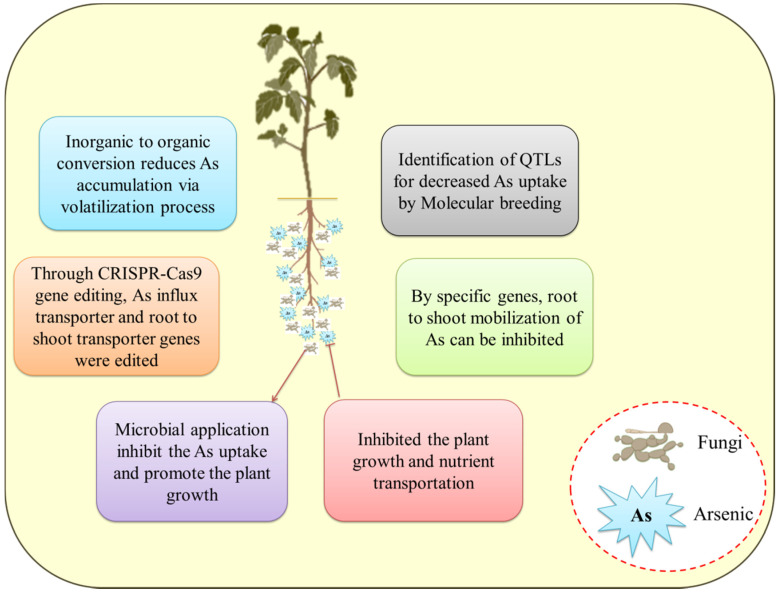
Arsenic reduction through biotechnological interventions in various crops.

To obtain As-free crop, As-resistant plant growth-promoting microbes have great potential in this regard because they are eco-friendly, cost-effective, and safe for crop production thereby reducing the As accumulation in plants (Figure 3). Apart from that, sprinkler irrigation methods are also used. The capacity of the great majority of higher plant species (about 90%) to associate with mycorrhizal organisms makes the spread of this method possible and practicable. In one investigation, numerous fungi isolated from contaminated site belonging to *Emericella, Fusarium*, and *Rhizomucor* sp. were shown to be capable of tolerating high concentrations of As. Some of these fungi were also able to improve growth and yield in crops by improving the soil physio-chemical properties and soil enzyme activity when crops were irrigated with sterile water containing As [117]. By lowering the accumulation of Zn in the wheat plant, another mycorrhizal fungus, *Funneliformis geosporum*, was able to enhance soil quality and boost the development and production of the wheat crop in Zn-polluted soil [131]. This study reveals that these native fungi boost plant development and crop production in agricultural regions that are heavily polluted with toxic metals, thereby removing toxic metals from the soil. Similarly, *Pleurotus ostreatus* also proved to be successful in removing the toxic metals from coal washery effluents, including, Pb, Zn, Cr, Co, Cu, and Ni [132]. Other fungi, including *Absidia cylindroslora, Fomitopsis meliae, Trichoderma ghanense,* and *Rhizopus microsporus*, were also able to withstand Cu, Cd, As, Pb, and Fe [66,133]. Nonetheless, scientists and researchers are also focusing on different genes that are related to As uptake, transport, and detoxification to understand their mechanism, isolate GEY and As-tolerant fungi, as well as generate As-free rice crops for human intake. To give resistance to toxic metals stress in natural habitats, fungi have evolved active defensive mechanisms such as biosorption, bioaccumulation, metal chelation, methylation, efflux transport, biostimulation, bioaugmentation, and biovolatilization. In the bioremediation of polluted regions, metal nanoparticle creation, and metal extraction from ores, the fungal resource has emerged as a distinct possibility. In Figure 3, various options for reducing the toxicity of As to plants are shown. Metal extraction and nanoparticle production capabilities of novel fungal strains are being investigated. The results of the fungal consortium are similarly impressive. Multiple metal-contaminated locations, on the other hand, are the best source for toxic metals-tolerant fungal isolation. Other contaminants found in these contaminated locations include nitrate, phosphate, sulphates, fluoride, pesticides, polyaromatic hydrocarbons, and others. Many fungal species (*Aspergillus ustus* and *Purpureocillium lilacinum*) have been shown to be capable of removing these contaminants in investigations [7,134,135,136]. As a result, using fungi to treat toxic metals and other contaminants together is a novel strategy with potential applicability in wastewater treatment. The use of harmful fungus can also be avoided, and the remediation efficacy of microbes can be enhanced, thus genetic engineering has a lot of potential in the future. Furthermore, the pathways that deal with fungus tolerance and toxic metals elimination produce unexpected results, but their full potential has yet to be realized. Since its implementation in various fields and quality production of the rice grain is a serious concern. With the usage of gene-editing tools CRISPR (clustered regularly interspaced short palindromic repeat)-Cas9, the crop production is upgraded and is helpful in the characterization of the genes.

## 5. Mechanism of Recombinant Yeast and Fungi-Induced Arsenic Remediation

**Figure 4:**
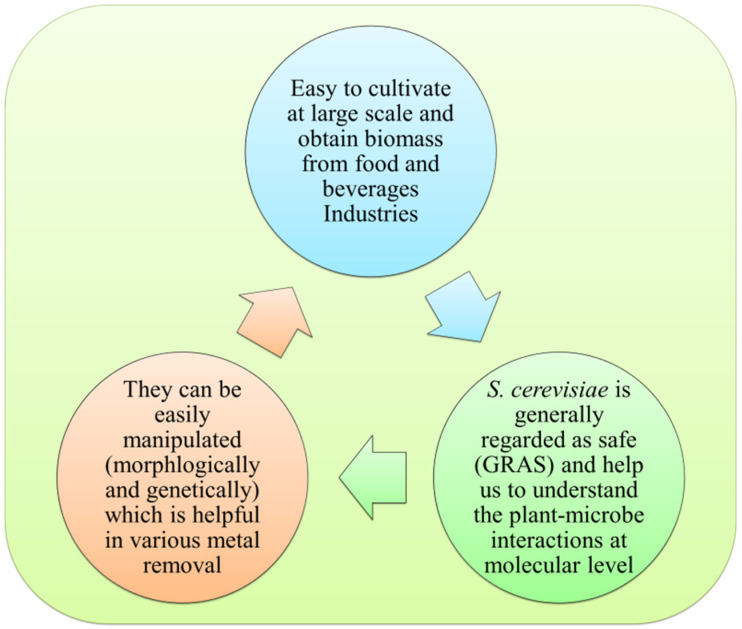
*Saccharomyces cerevisiae*-mediated plant growth promotion and arsenic remediation.

Metal resistance has developed in microbes as a result of their constant exposure to harmful metals since life began at least 3.5 billion years ago. Metal–fungus interaction is influenced by a number of parameters, including the kind and concentration of metal, the organism, and the nature of the polluted environment. Fungi have developed both internal and external mechanisms to counteract the harmful effects of unwanted metals. Metal uptake or metal sequestration mechanisms by *S. cerevisiae* are of two kinds: (i) active mode by living cells and (ii) passive mode by inactive/dead cells of *S. cerevisiae*. Active mode is associated with metal transport and its deposition and is metabolism-dependent. Passive mode is energy freelance, principally through functional groups of the material, containing the cell and significantly cell wall [9]. Many yeasts have conjointly reported exhibiting plant growth-promoting (PGP) traits such as the production of phytohormone, solubilization of PO_3^−^_, inhibition of numerous pathogens, oxidation of S and N, and mycorrhizal root colonization promotion [137]. Genetically modified yeast (GMY) may thus play an important role in plant growth promotion even in the presence of toxic metals and As (Figure 4). Yeast containing arsenic methyltransferase (*WaarsM*) gene consecutively methylates the toxic inorganic As to less toxic pentavalent methylated arsenicals such as TMA oxide, DMAV, and MMAV [93]. The biovolatilization of As leads to forming volatilized TMAIII and will take away the As from contaminated sites, thereby providing a possible strategy for lowering the As from soil. This mechanism was used to enhance As volatilization by over-expressing *arsM* gene in several micro-organisms [138]. The Bioremediation of As-polluted soil using GEY (genetically engineered yeast) could be a preferable option as it reduces the As content which is present in the soil, and promotes plant growth by lowering the As accumulation in the soil. It has been stated that *S. cerevisiae* expressing the *WaarsM* (*Westerdykella aurantiaca*) gene lowers the As accumulation by biovolatilization and methylation in an As-rich environment [110,139]. However, the influence of exogenous yeast (with PGP abilities) on As volatilization, on the other hand, has never been investigated. A study by [139] examined the capability of GEY in promoting plant growth in the presence or absence of As, as well as the effect of GEY inoculation on seed growth.

Numerous studies have demonstrated that certain fungi may accumulate and eliminate various pesticides from the environment [140,141,142,143]. *Aspergillus tamarii* and *Botryosphaeria laricina* isolated from the agricultural field previously exposed to endosulfan were tolerant to endosulfan and were able to degrade the toxicant and its harmful metabolites such as endosulfan sulfate, alpha endosulfan, and beta endosulfan by using them as a source of carbon and energy [144]. A unique strain of *A. glaucus* was also shown to be capable of metabolizing fipronil and its byproduct, fipronil sulfone, in another investigation [145]. Some aquatic fungi such as *Mucor hiemalis, M. hiemalis* EH5, and *M. rouxii* can accumulate and degrade cyanotoxins as they are tolerant to oxidative stress [146,147,148]. Recently, a newly isolated yeast called *Diutina rugosa* has played a significant role in degrading the indigo dye from soil polluted with wastewater [149]. A thorough research of As tolerance in fungi revealed the involvement of extracellular systems such as metal chelation and cellular binding, which prevent metal ions from entering the cell core. Metal ions were conjugated with fungal biomolecules such as proteins and chemical ligands in intracellular methods. As a result, we divided the fungus’ defensive mechanisms into different subheadings, including (i) Biosorption and Bioaccumulation; (ii) Chelation of metals; (iii) Cell surface precipitation; (iv) Bioaugmentation and biostimulation; and (v) Biovolatilization and Methylation.

### 5.1. Biosorption and Bioaccumulation

**Figure 5:**
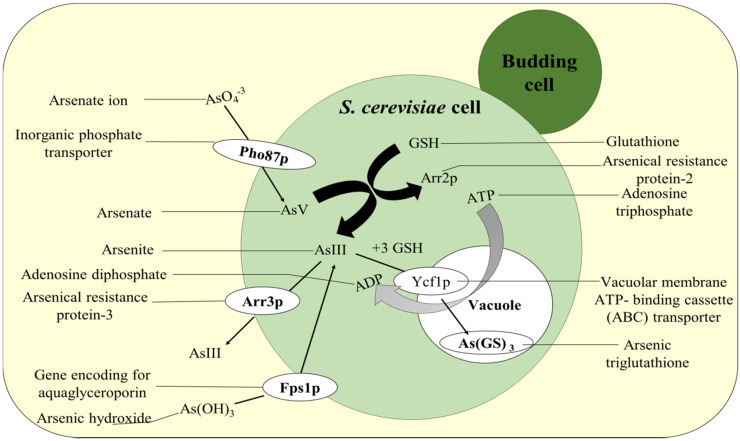
Arsenic detoxification mechanism used by *Saccharomyces cerevisiae*. In *S. cerevisiae*, AsIII and AsV are transported by the cytoplasmic arsenate reductases ArsC and Arr2p. Arr3p is a potential-driven arsenite efflux protein that combines with ArsA to produce an ATPase (in bacteria). Several glycerol proteins (GP) also transport arsenite, including *GlpF* and *Fps1p*. *PHO87p* is an uptake transporter that transports phosphates (and arsenates). In yeast cells, *Ycf1p* is a transporter of As(III)-3 GSH adducts into the cellular vacuole compartment, which functions as an ATPase. GSH—glutathione.

In biosorption, the extra-cellular sequestration of toxic metals occurs, thereby preventing their entry inward the fungal cells and thus maintaining the metal homeostasis. Many fungal cells adsorbed As by forming chemical bonds with cell surface molecules that have certain functional groups as: glycoprotein, polysaccharide, and glycolipids, among others. *A. niger* has the ability to absorb As in high concentration due to the prevalence of the functional group in their cell wall. It was analyzed that *A. niger* can remove more than 90% As at all tested concentrations [17]. This involves the combination of several processes such as entrapment, chelation, micro-precipitation, and complexation as a remedial activity from contaminated sites [8]. The extreme biosorption value was documented around 108.08 mg g^−1^ at As concentration of 600 mg L^−1^ [150]. The *Aspergillus candidus* was found to remove the highest amount of As after three days of growth in presence of 25 mg L^−1^ (trivalent) and 50 mg L^−1^ of pentavalent arsenic [151]. For the transportation of AsIII and AsV inside the yeast cell, cytoplasmic arsenate reductase proteins such as bacterial arsenate reductase (ArsC) and yeast arsenate reductase (Arr2p) are involved. The Arr3p (protein product of *ARR3*) are mainly potential driven membrane AsIII efflux protein which transports the AsIII formed in the *S. cerevisiae* cell to outside [10]. Apart from this, *Fps1p* are the glycerol transport proteins which are also responsible for AsIII transportation in the reverse route (Figure 5). The *PHO87p* forms the potential-coupled PO_3^−^_ uptake transporter [152]. The AsIII GSH is carried by vacuolar ABCC protein yeast cadmium factor 1 (*Ycf1p*) inside the vacuole of the yeast cell. This transporter protein also functions as ATPase [153].

Bioaccumulation is metabolically an active process which is accomplished by living cells [154]. It is well-defined as the cultivation of As-tolerant fungi in the presence of sorbate [154]. It elucidates the intracellular accumulation of sorbate, which is a non-equilibrium process that requires the metabolic activity of the cells. Different metabolic processes such as biomineralization and biotransformation are utilized for the removal of toxic ions [155] such as lead (Pb), Manganese (Mn), and Iron (Fe) [156]. The ability for the effective accumulation of As from arsenopyrite by *Serpula himantioides, A. niger*, and *Trametes versicolor* is well documented by Al-Makishah et al. [157]. This is due to their specific metal-binding proteins and peptides as MT. Additionally, it was also confirmed by Mukherjee et al. [98] that *A. niger* has high AsV uptake capacity and results displayed that As content in fungal biomass is elevated with an increase in initial AsV concentration. In bioaccumulation, pollutants are also transported intracellularly across the cellular wall and cell membrane [158]. Higher As bioaccumulation and biovolatilization have been observed in seven fungal strains—*Fusarium* sp. FNBR_LK5, FNBR_B7, and FNBR_B3; *Emericella* sp. FNBR_BA5; *Aspergillus oryzae* FNBR_L35; *A. nidulans* FNBR_LK1; and *Rhizomucor variabilis* sp. FNBR_B9 [117]. Many of the fungi listed above have the ability to remediate toxic metal pollutants. For instance, *A. oryzae* could be also more operative for AsV remediation from aqueous solution with the help of their functional groups such as HO^−^;, NH_2_, and carboxyl groups which are present on the fungal mycelia. The maximum tolerant concentration of *A. oryzae* towards AsV has reached 5000 mg L^−1^ [159].

**Figure 6:**
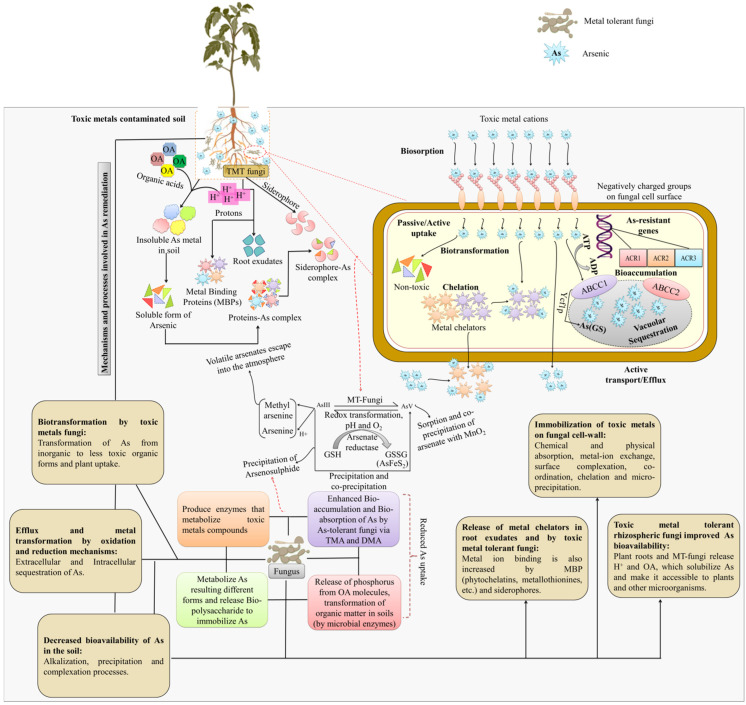
Mechanism of arsenic tolerance in fungi at the cellular level. Organic acids and protons are produced by MT-fungi, which transform the insoluble form of As metal into a soluble form. They were then able to bind MBPs, generating a complex that plants could easily absorb. Redox conditions influence the equilibrium between AsV and AsIII in the circulating solution of soil. Once inside the plant, arsenate reductase reduces AsV to AsIII, while GSH is oxidized to GSSG. In yeast and several fungal cells, the Acr2p arsenate reductase uses glutathione oxidation to convert AsV to AsIII (GSH to GS). Two routes for eliminating cellular AsIII include extrusion via the plasma membrane carrier Acr3 or conjugation to glutathione (As(GS)_3_), which is sequestered into vacuoles by the ABC transporter *Ycf1p*. As—Arsenic; TMT—Toxic metal tolerant; OA—Organic acids; OM— Organic matter; H^+^—Protons; MBPs—Metal binding proteins; AsIII—Arsenite; AsV—Arsenate; GSH—Glutathione; GSSG—Glutathione disulfide; AsFeS_2_—Arsenic pyrite; TMA—Trimethyl arsine; DMA—Dimethylarsinic acid; ATP—Adenosine triphosphate; ADP—Adenosine diphosphate; *ACR*—Arsenic compounds resistance; ABCC—ATP binding cassette subfamily C transporter; *Ycf1p*—Yeast cadmium factor-1.

For As tolerance and detoxification, the eukaryotic model organism *S. cerevisiae* has been intensively researched. The gene cluster *ACR1*, *ACR2*, and *ACR3* give As tolerance in yeast. *ACR1* encodes a putative transcription factor that controls the transcription of *ACR2* and *ACR3*, potentially by detecting cellular As levels directly. *ACR2* encodes an arsenate reductase, while *ACR3* encodes an AsIII-efflux transporter expressed in the plasma membrane (Figure 6). Consequently, the gene cluster acts as a detection, reduction, and efflux mechanism for As. However, vacuoles in yeast provide a second mechanism for detoxification: cytosolic AsIII complexed with glutathione may be sequestered into this compartment via an ABC-type transporter called ycf1, which also transports conjugates of other toxic chemicals [160].

### 5.2. Chelation of Metals

Another toxic metals resistance technique is metal ion detoxification or metal chelation. In response to toxic metals stress, fungi produce chelating molecules, which bind to metal ions and minimize detrimental effects. Metal detoxification agents include –SH containing compounds, MTs, heterogeneous and homogeneous proteins, organic acids (citric acid, oxalic acid), and peroxidases [161]. When these chelating molecules come into contact with hazardous metal ions, they generate complex non-toxic metal forms that are sequestered in various cellular organelles (Figure 6). In significantly polluted soil, studies show an increase in microbial metabolism. This is because the energy requirement of microbial functional groups involved in metal ion absorption and chelation has increased [162,163,164]. In the next parts of this review, the functional role of several metal chelating agents has been detailed in depth.

#### 5.2.1. Organic Acids

In reaction to metal stress, several fungi release organic acids, which aid in the solubilization of metal ions and the formation of metal oxalates. Acids are used to detoxify metals both extracellularly and intracellularly. Copper (Cu) and lead (Pb) have been found to be detoxified by oxyalic and citric acids released by *A. niger, Penicillium* sp., and *Rhizopus* sp. (Figure 6). A mycorrhizal fungal species, *Rhizopogon roseolus*, has also been shown to create oxalic acid in considerable amounts in reaction to toxic metals [165]. Certain species of wood rooting fungi, such as *Bjerkandera fumosa*, *Phlebia radiata, T. versicolor*, and *Fomitopsis pinicola,* have been found to use oxalic acid as a mechanism of surviving metal stress [166].

#### 5.2.2. Compounds That Chelate Metals

Metal ion detoxification is also aided by proteins, peptides, enzymes, and certain –SH-containing compounds. In reaction to Pb, proline, malondialdehyde, and catalase enzymes are known to be generated [167]. To withstand AsV toxicity, the *Aspergillus* sp. P37 strain employs reduced glutathione (–SH group compounds)-like substances. With decreased arsenate, the molecule forms an As(GS)_3_ complex, which accumulates in the vacuoles (Figure 6). The vacuole’s acidic pH aids in stabilizing the entrapped As(GS)_3_ [105]. In the fungus *Paxillus involutus*, –SH-containing GSH is also engaged in the non-enzymatic detoxification of H_2_O_2_ and scavenges O_2_ radicals formed in response to cadmium (Cd) stress [168]. Melanin is a kind of fungus that produces chelators in reaction to toxic metals. They are made up of peptides, carbohydrates, fatty acids, phenolic units, and aliphatic hydrocarbons, and hence effectively bind to metal ions, resulting in the formation of electron-dense granules [169]. Gadd and de Rome [170] found that melanin from the fungi *Cladosporium resinae* and *Aureobasidium pullulans* had stronger Cu ion sorption than the entire biomass in research. Metallothioneins are a kind of metal chelator made up of –SH groups that are formed in response to metal stress in fungi, algae, and plants. Morselt et al. [171] were the first to report on the role of MT in copper and zinc resistance in the fungus *Pisolithus tinctorius*. Later, thiol molecules (PCs, MTs, and GSH) were shown to be involved in Cd detoxification in the ecto-mycorrhizal fungus *P. involutus*. Treatment with Cd resulted in an increase in glutathione and g-glutamylcysteine levels [172].

#### 5.2.3. Metal Exclusion through Efflux Transport

The efflux mechanism is critical for controlling metal concentrations in the cell interior. Microorganisms that take up both necessary and non-essential metals have a variety of non-specific transport mechanisms. When metal ions reach a concentration that is potentially hazardous to bacteria and fungi, however, certain active efflux mechanisms help to expel the metals. Under high metal stress circumstances, the fungus is known to use both metabolism-dependent and metabolism-independent mechanisms to survive [173]. The numerous metal-binding sites in fungal cells are summarized in (Table 2).

Metal resistance in microorganisms is known to be mostly dependent on active transport or efflux mechanisms. Metal ions that have collected in the cytoplasm are expelled from the cell. This method is usually used by bacteria, but it has also been reported that some As-resistant fungi employ it as a defensive mechanism. Metal ion toxicity is also reported at both the cellular and molecular levels. Fungi’s cellular defense systems may be divided into two categories: extracellular and intracellular. The former prevents metal ions from being taken up and internalized, whereas the latter lowers the harmful effects of imprisoned metals by attaching to biomolecules or efflux channels. To combat AsV toxicity, *Aspergillus* sp. P37 uses an extracellular efflux mechanism in addition to intracellular AsIII buildup. The fungus converts AsV to AsIII, then excretes the reduced arsenate from the cell [105]. In the wild type arsenic-tolerant fungus *A. niger*, a similar mechanism has been postulated [98]. A comparison of an AsV-resistant fungus *Hymenoscyphus ericae* (isolated from polluted mining sites) and a non-resistant *H. ericae* revealed that the resistant strain had a faster efflux transport mechanism than the non-resistant strain [181]. Arsenic absorption occurred through passive diffusion in both cases and followed comparable uptake rates; however, the AsV-resistant type lost 90% of the ingested arsenate. In comparison, just 40% of the non-resistant strain was removed.

### 5.3. Cell Surface Precipitation

The cell wall tends to be the primary cellular structure approach in contact with various metal ions, excluding a potential prevailing extracellular layer that is principally associated with microbial cells. The primary mechanisms of metal-uptake by cell-wall are through stoichiometric interaction between various functional groups of cell-wall such as NH_2_, R-COOH, PO_3^−^_, and phosphodiester. At present oxidation, reduction, adsorption (by van der Waals force/electrostatic interaction), and ion exchange have been put forward to explain the metal uptake by an organism [182,183]. The detoxification of metal ions may be understood by oxidation, reduction, demethylation, and methylation. Rosen [184] show that one mechanism of AsV detoxification was the reduction of AsV to AsIII, catalyzed by an enzyme called arsenate reductase. Several genes concerned within the detoxification/uptake or tolerance towards metal ions have also been recognized [184]. For instance, the *S. cerevisiae* Arr4p plays a critical role within the tolerance to several metal ions such as cobalt (Co^2+^), chromium (Cr^3+^), arsenite (As^3+^), arsenate (As^5+^), copper (Cu^2+^), and vanadate (VO_4_^3−^) [185].

### 5.4. Bioaugmentation and Biostimulation

The process of adding cultured microorganisms into the contaminated site for removing the groundwater contamination and biodegrading specific soil is known as bioaugmentation. It has been well documented that the GEY cells *S. cerevisiae* (expressing *A. thaliana* Phytochelatin Synthase (*AtPCs*)) can accumulate and tolerate a huge amount of As [186]. Tsai et al. [113] expressed two different genes, i.e., cysteine desulfhydrase and AtPCs in GMY and control yeast cells and showed an elevated level of As accumulation. Therefore, these GMY cells could be used to remove the arsenic from the environment. Biostimulation is the introduction of enhanced nutrients to a polluted site in order to boost the efficiency of bioremediation by stimulating the microbiological development of indigenous bacteria. The study analyzes the reduction of As accumulation in numerous parts of rice crop such as shoots, roots, grain, and husks, with the inclusion of Kaolin, thereby reducing the As accumulation in rice crops [187]. The application of both bioaugmentation and biostimulation at the same time produces better outcomes than individual use due to a synergistic impact. Analysis showed that the use of genetically engineered bacteria (*P. putida* KT2440), with 5% of rice straw, revealed the maximum efficiency of As volatilization, thereby providing a very helpful model/system for overcoming the As contamination in agrarian soil which ultimately reducing As accumulation in rice grains [188]. Similar results have also been reported using *Brevundimonas diminuta* (NBRI102) bacterial strain [189].

### 5.5. Biovolatilization and Methylation

Biovolatilization is an enzymatic process in which the metalloids are converted into their volatile derivatives through biochemical reactions [190]. The conversion of the toxic form of As into gaseous form using methylating fungi is known as methylation. Intracellular As absorption and binding of AsV onto fungal cells might be the viable explanation for rhizospheric As immobilization thereby lowering the plant As uptake [190]. *Aspergillus glaucum, Scorpularipsis brevicaulis, Candida humicola, Penicillium gladioli*, and *Fusarium* sp. can biomethylate arsenic into volatile TMAs using inorganic As [17]. The speciation of As in fungal biomass has revealed that *Penicillium* sp. presents higher methylated As-species than any other fungi. Based on this, it has been concluded that *Penicillium* sp. was the fungus showing the highest ability to volatilize the TMAs, followed by *A. niger. Aspergillus niger* has presented the maximum inorganic As biomass accumulation, the chosen form for chelation [17].

## 6. Conclusions and Future Perspectives

Toxic metals are the major contributors to environmental pollution that have serious consequences for human health as well as the environment. Therefore, mycoremediation is an effective and sustainable method for treating toxic metals contaminated areas that has many advantages over conventional treatment technologies. Numerous fungal isolates such as *Aspergillus, S. cerevisiae,* and *Penicillium* can grow at high As concentration and hence this fungal biomass is efficient for the mycoremediation of As contaminant. *S. cerevisiae* is a promising biomaterial used for various metal removal due to its distinctive characteristics and has received increasing attention throughout the past decades. In the bioremediation of polluted environments, the fungal resource has emerged as a distinct possibility. Thus, fungal biomass plays an essential role in biovolatilization, bioaccumulation, biosorption, metal chelation, cell surface precipitation, and methylation, thereby helping plants to sustain themselves in As-contaminated soil.

Arsenic toxicity in various crops is a matter of serious concern for the well-being of humankind. On the whole, As exposure, deregulates various cellular processes such as epigenetic regulation, DNA repair, apoptosis resistance, and normal gene expression. All of them are vital in carcinogenesis evolution, so targeting these signaling pathways would possibly provide a therapeutic approach/alternative for the treatment and prevention of chronic As exposure-related cancers. In plants, As exposure led to the induction of ROS which led to oxidative stress to the plants. This oxidative stress is countered into the vacuole via –SH rich compounds such as PCs and GSH. To combat As deposition in plants, a number of plant-based methods and agronomic strategies are now available. A growing body of research demonstrates that genetic manipulation approaches can improve a plant’s As-complexation capacity and raise its arsenic tolerance, and Table 3 highlights these attempts.

The increasing understanding of the properties of materials, such as biochar, as a function of treatment conditions, can enable more focused usage and, more importantly, reduce reliance on the high variability of organic composted and non-composed resources [204,205]. The outcomes that are discussed in this review could be used to overcome the adverse effect of As and reduce the As accumulation in rice grains by gene editing, molecular breeding, and transgenic approaches. The disposal or reuse of bedding material, as well as the recovery of As from it, is a new problem that must be addressed in the future to ensure the long-term viability of wetland systems. Moreover, the presence of numerous signaling pathways, as well as their interactions, adds to the complexity of the cell-ROS signaling and heavy-metal axis, which requires further investigation.

On the basis of the information already available, the following next study directions are suggested: (i) Further studies are needed to investigate how toxic metal tolerant fungi affect plant biochemistry; (ii) the most crucial aspect of future research on metal toxicity tolerant fungi in plant biology should concentrate on fully utilizing the role of these fungi and even PGPRs (co-inoculation of beneficial mycorrhizal fungi and PGPRs) in conferring tolerance in plants against metal toxicity stress and thus their involvement in environmental remediation; (iii) inoculation with potential fungi will likely have the greatest beneficial effects in poor soils because competition for limited resources is crucial and metal-tolerant fungi are also prone to environmental stresses; (iv) with our agricultural crops being increasingly exposed to abiotic stresses, including metal toxicity, it is imperative to optimize inoculants for agricultural use in tandem with ongoing efforts to develop stress-tolerant crops; (v) research needs to be conducted to determine whether metal toxicity tolerant fungi can reduce toxic metal accumulation in plants irrigated with industrial or municipal wastewater; (vi) in order to prevent metal buildup in plants growing in soils that are polluted with many toxic metals, it is advised to investigate microorganisms that are resistant to a variety of toxic metals; (vii) Finding endophytic microbes that are effective in accumulating toxic metals solely in the roots of plants grown in soils polluted with metals is necessary; (viii) it is generally known that field outcomes might vary from those attained in a greenhouse or under in vitro circumstances. Further field research is thus necessary to evaluate the potential of such methods for agricultural production systems as well as their effects on crop development and soil behavior; (ix) in order to choose which type of microbe is best to utilize with which plant in a certain circumstance, it is crucial to understand the processes of metal toxicity-tolerant mycorrhizal fungus. Therefore, it is important to research how various plants in various environments respond to mycorrhizal fungi that are resistant to metal toxicity. Does the observed mycorrhizal fungi-mediated metal toxicity-tolerance fluctuate with plants’ physiological status? Do special mycorrhizal fungi exist to accumulate a special toxic metal in themselves forever? Which toxic metal may be reverted more frequently, and by which fungus genus and species? Is it feasible for the plants’ physiological status and environmental factors to alter without releasing the metals that mycorrhizal fungi have taken in? Taken together, it is essential that well-designed, large-scale and long-term field tests be conducted to assess the feasibility of application of metal toxicity tolerant mycorrhizal fungi for relieving metal toxicity stress in the field. Therefore, we can conclude that usage of myco-remediation or fungi as a consortium is used as an alternative and sustainable tool for the removal of As contamination. It would be a beneficial approach towards sustainable agriculture, thereby meeting the sustainable development goals and making the crop sustain under abiotic metal stress. To overcome the health consequences caused by As contamination, an in-depth study concerning cell-ROS signaling heavy-metals would be useful in understanding the further secrets about how cells transform themselves from a healthy form into a pathological form following toxic metal exposure.

## Figures and Tables

**Table 1:** Mode of action of fungal isolates in arsenic contaminated area.

Fungal Isolates	Time Taken in Remediation Process	Effect of Fungal Isolates on Soil	Mechanism of Action	Removal Efficiency (%)	References
A consortium of *Ascomycota* and *Basidiomycota*	7 days	Enhanced soil quality, increased enzymatic activity	Volatilization/biomethylation	31–77%	[8]
*Trichoderma* sp. *MG* and *H. annuus*	6–8 days	Improves the soil enzymes activity, and also plant–fungi partnership for enhanced bioremediation of As	Bioaccumulation	In shoot 67%, in roots, 55%,	[116]
*P. brevicaule* and *Aspergillus oryzae*	21 days	Improves the agricultural soil	Bioaccumulation and volatilization	82% and 6.4 mg kg^−1^	[117]
*Lasiodiplodia* sp. *and Mycelia strain(FA-13)*	21 days	Enhances soil quality	Bioaccumulation/biosorption	65.81%	[14]
*P. janthinellum*	5 days	Improves the soil enzymes activity, and also plant–fungi partnership for enhanced bioremediation of As	Bioaccumulation	87.0 µg g^−1^	[118]

**Table 2:** Toxic metals binding to fungal cells are aided by many cellular locations.

Location	Involved Functional Groups in Metal Uptake	References
Cell organelles	Cot1/Zrc1 transporters transfer to vacuoles. e.g., *S. cerevisiae*	[174]
	Formation of vesicles (Zincosomes) e.g., *S. cerevisiae*	[175]
Cytoplasm	Metal oxalates e.g., *Beauveria caledonica*	[176]
	Precipitate of Cu and AsV e.g., *Aspergillus foetidus*	[177]
	PO_3^−^_ precipitates e.g., *Penicillium* sp. *PT1*	[178]
Extracellular	Electronegative interaction with spores’ exterior surface. e.g., *Mucor hiemalis*	[65]
	PO_3^−^_ binding ligands e.g., *M. hiemalis*	[65]
	–OH and –C=O groups e.g., *Penicillium chrysogenum XJ-1*	[179]
	CH-OH and OH/NH_2_ functional groups e.g., *A. fumigates*	[180]

Cot1/Zrc1: Cobalt uptake protein/zinc ion trans-membrane transporter; PO_3^−^_: Phosphate; –C=O: Carbonyl group; NH_2_: Amino group; OH: Hydroxy group.

**Table 3:** List of plants with altered As tolerance acquired either by expression of foreign genes or through overexpression of wild type genes.

Genes	Products	Gene Source	Principal Outcomes	References
*OsNRAMP1*	Natural resistance-macrophage protein transporter	*O. sativa*	The transgenic line’s shoots and roots had a two-fold greater concentration of As than the WT.	[191]
*PvTIP4;1*	Transporter of the TIP	*Pteris* *vittata*	Significant increase in As accumulation was linked to increasing vulnerability to As stress in transgenic lines as compared to the WT.	[192]
*PvACR3;1*	Arsenic compound resistance 3 (AsIII antiporter)	*P.* *vittata*	In comparison to the WT line, the transgenic line displayed increased As retention in roots and decreased As translocation to shoots.	[193]
*AtABCC1* and *AtPCS1*	ATP binding cassette subfamily C transporter and PC synthase	*A. thaliana*	Overexpression of both genes at the same time resulted in enhanced As complexation by PCs and improved transport to the vacuole.	[194]
*AtPCS1*	PC synthase	*A. thaliana*	The transgenic line has a much higher tolerance to As when compared to the WT.	[195]
*AtPCS1*	PC synthase	*A. thaliana*	Arsenic resistance was observed in plants overexpressing *AtPCS1* from a strong constitutive *Arabidopsis* actin regulatory sequence (A2).	[196]
*AtPCs1*	PC synthase	*A. thaliana*	Plants that overexpressed *AtPCS1* in the cytoplasm were more resistant to As than WT plants. When *ATPCS1* was directed to the chloroplast, the effects were the total opposite.	[197]
*ACR2*	Arsenate reductase	*A. thaliana*	The transformant has a higher tolerance to As and accumulates less of it than the WT.	[198]
*GSH1* and *AsPCS1*	γ-glutamylcysteine synthetase and Phytochelatin synthase	*S. cerevisiae* and *Allium sativum*	Tolerance to As has increased in both single-gene and double-gene transformants. Dual gene transformants outperform single gene transformants.	[199]
*GSH1* and *AsPCS1*	γ-glutamylcysteine synthetase and Phytochelatin synthase	*Escherichia* *coli constructs*	Increased resistance to As in transgenics, but with a larger ability for accumulation than the WT.	[200]
*AsPCS1* and *CePCS1*	Phytochelatin synthase	*A. thaliana* and *Caenorhabditis elegans*	Increased in the lines that co-express both genes, there is As-tolerance.	[201]
*AtPCS1*	PC synthase	*P. vittata*	Enhanced As tolerance in transgenic lines as a result of increased AsV reduction and improved AsIII efflux via aquaglyceroporin regulation.	[49]
*OsGrx_C7* and *OsGrx_C2.1*	Glutaredoxin	*O. sativa*	In comparison to the WT, transgenic expression of *OsGrxs* resulted in much lower As accumulation in seeds and shoots, as well as higher As tolerance.	[202]
*CrarsM*	SAM-methyltransferase	*Chlamydomonas reinhardtii*	The transgenic line had a considerable capacity to methylate As, however, this was accompanied by increased vulnerability to AsIII.	[203]

TIP—Tonoplast intrinsic protein; PC—Phytochelatin; WT—Wild type; AsIII—Arsenite; AsV—Arsenate.

## Data Availability

The data presented in this study are available in the article.
